# Adaptive evolution of *Candida albicans* through modulating TOR signaling

**DOI:** 10.1128/mbio.03947-24

**Published:** 2025-03-04

**Authors:** Yaling Zhang, Lianjuan Yang, Youzhi Zhao, Kang Xiong, Hao Cui, Tianxu Wang, Xiaoping Liu, Chang Su, Yang Lu

**Affiliations:** 1 Hubei Key Laboratory of Cell Homeostasis, College of Life Sciences, TaiKang Center for Life and Medical Sciences, Wuhan University, Wuhan, China; 2 Shanghai Dermatology Hospital, School of Medicine, Tongji University, Shanghai, China; 3 Hubei Key Laboratory of Cell Homeostasis, College of Life Sciences, Wuhan University, Wuhan, China; The University of Texas Health Science Center at Houston, Houston, Texas, USA

**Keywords:** *Candida albicans*, TOR, commensal fitness, adaptive evolution, environmental adaptation

## Abstract

*Candida albicans* stably colonizes humans but is a major fungal pathogen that occupies a wide range of divergent niches within the host. Rapid and effective adaptation to dynamic and contrasting host niches is associated with its pathogenicity. Recent studies have focused on genome evolution implicated in adaptive processes. Here, we demonstrate that modulation of TOR signaling is a mechanism underlying adaptive evolution in *C. albicans*. Clinical isolates of *C. albicans* exhibited enhanced commensal fitness in competition with the lab reference strain SC5314, which could be attributed to the diminished GlcNAc-responsive hypha-associated transcription in the gut. *In vitro* passaging of clinical isolates confers a reduction in TOR signaling, which is detrimental to fitness attributes in evolved strains, including stress response, antifungal drug tolerance, as well as *in vivo* commensal fitness and invasive infection. This phenomenon is observed independent of strain background and passaging environment. Importantly, inhibition of TOR signaling by rapamycin suppresses the fitness advantage observed in clinical isolates relative to their *in vitro* passaged derivatives. Thus, *C. albicans* undergoes rapid evolution via modulating TOR signaling that enables this fungus to adapt to diverse host niches.

**IMPORTANCE:**

Pathogens must be proficient at adapting to their surroundings to survive in the face of a changing microenvironment in the host and cause disease. This is particularly important for commensal-pathogenic organisms such as *C. albicans* as this fungus colonizes and infects mammalian hosts. Previous studies have focused on genome evolution such as aneuploidies, accumulation of point mutations, or loss of heterozygosity. Here, we demonstrate that *C. albicans* undergoes rapid adaptive evolution via modulating the TOR pathway. Alterations in TOR activity underlie some evolved traits with important consequences for both host adaptation and pathogenicity in *C. albicans*. Such mechanisms of adaptive evolution may be exploited by other organisms.

## INTRODUCTION

*Candida albicans* is a widespread human commensal of the skin, oral cavity, and gastrointestinal (GI) and genitourinary tracts. However, overgrowth of these host niches can result in debilitating mucosal infections in healthy individuals and life-threatening systemic disease in immunocompromised patients, making *C. albicans* an opportunistic pathogen of significant clinical importance (1–4). Rapid and effective adaptation to dynamic and contrasting niches, which enhances the fitness of the fungus, is often as essential for pathogenicity as virulence factors (5–7). From the fungal perspective, it is clear that *C. albicans* can adapt effectively to diverse host niches.

Studies in evolved isolates have implicated multiple mechanisms in host adaptation, including genome evolution such as aneuploidies (8, 9), accumulation of point mutations, or loss of heterozygosity (LOH) events (10–14). For example, although a handful of studies suggest that Chr 7 trisomy in microevolved isolates confers a fitness benefit in murine gut (15), most of the isolates with increased commensal fitness do not acquire aneuploidies and vary in evolution patterns (10, 11). Therefore, it is of great interest to explore alternative mechanisms that underlie adaptive evolution in this important commensal-pathogenic fungus.

Being a successful commensal and pathogen, *C. albicans* commensalism has evolved with retention of fungal virulence traits. The transition from yeast to hyphal cell morphology is central to *C. albicans*’ pathogenic potential (16, 17), whereas the hypha-associated transcription inhibits commensal fitness (18). N-acetylglucosamine (GlcNAc) is a component of the peptidoglycan of bacterial cell walls and the extracellular matrix glycosaminoglycans of animal cells (19). Our recent study demonstrated that GlcNAc is a major inducer of hypha-associated transcription in the gut, which represents the key determinant for commensal-pathogenic equilibrium (20). However, the impact of GlcNAc sensing in driving the adaptive process of *C. albicans* is less understood.

In this study, we demonstrate that *C. albicans* undergoes adaptive evolution through modulation of the target of rapamycin (TOR) pathway. By passaging clinical isolates under *in vitro* conditions, evolved isolates produced a commensal defect in comparison to their related parental strains. This might be attributed to a higher hypha-associated transcription in these evolved cells in GlcNAc and during gut colonization. Moreover, *in vitro* evolution conferred a reduction in fitness in organs during *in vivo* infection. Importantly, the fitness advantage exhibited in clinical isolates relative to their *in vitro* passaged derivatives can be suppressed upon rapamycin addition. Our study therefore reveals that high TOR signaling facilitates *C. albicans* adapting to host niches.

## RESULTS

### Clinical isolates of *C. albicans* outcompete SC5314 during gut colonization

**Fig 1:**
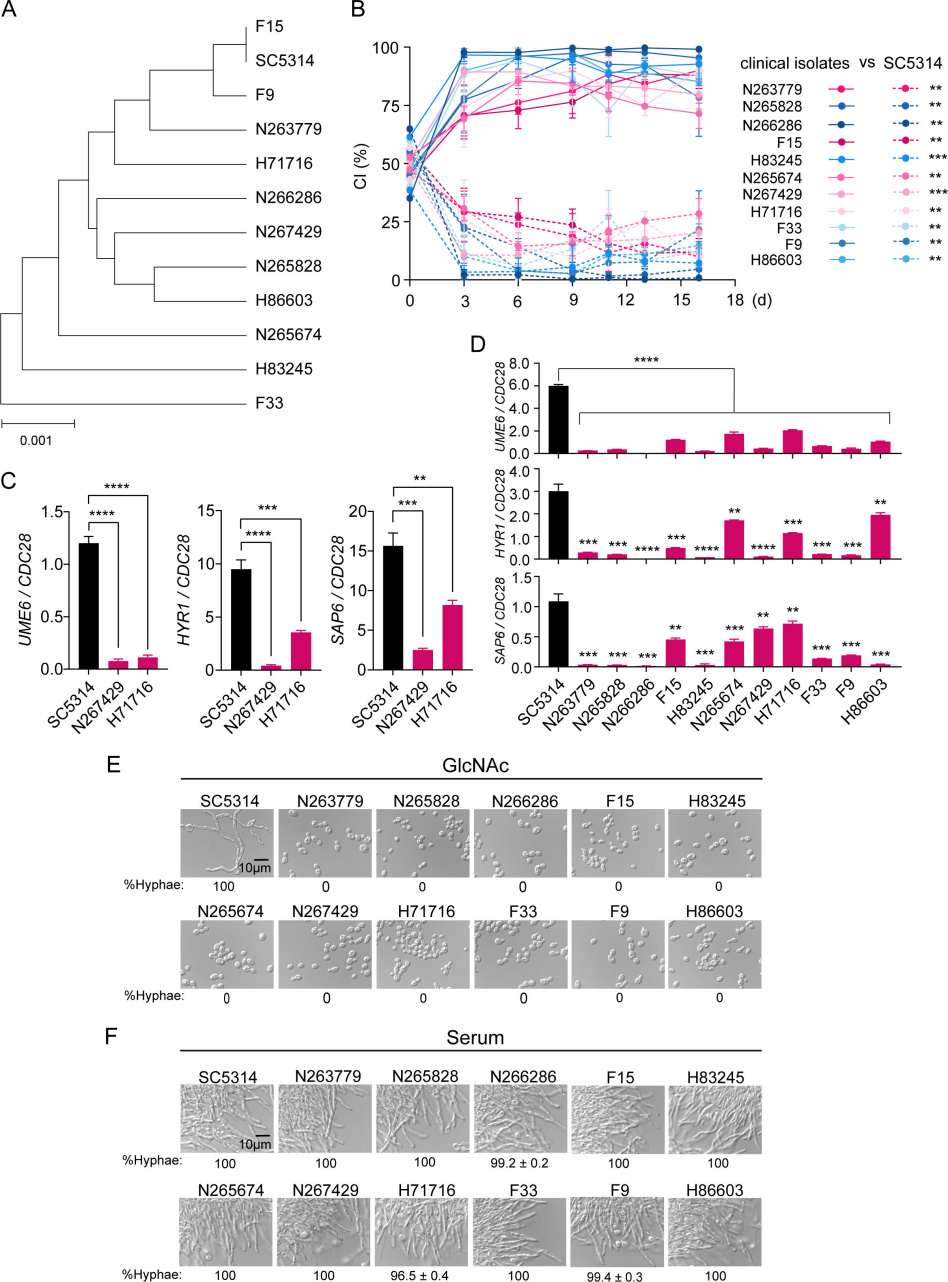
The enhanced commensal fitness in clinical isolates of *C. albicans*. (**A**) Genetic diversity inferred by UPGMA. The analyses were based on the sequencing results of seven housekeeping genes and performed using MEGA11 software. (**B**) Commensal competition between clinical isolates (solid line) and the lab reference strain SC5314 (dotted line). BALB/c mice (6–8 weeks old) were garaged with 1:1 mixtures of a nourseothricin-resistant strain and an unmarked strain. Mice were treated with antibiotic water (streptomycin, 2 mg/mL; penicillin, 0.97 mg/mL) for 3 days prior to inoculation and throughout the commensal competition experiment to remove bacterial species. Relative abundance of strains was monitored by collecting fresh fecal pellets and plating homogenates on YPD plates supplemented with or without 200 µg/mL nourseothricin. The competitive index (CI) was shown as the proportion of the indicated strain to the total. *n* = 3 mice housed separately. Data are presented as the mean ± SD. Significance was determined using paired two-tailed Student’s *t*-test. (**C**) qRT-PCR analysis of *UME6*, *HYR1*, and *SAP6* in SC5314 and indicated clinical isolates during commensal growth. BALB/c mice were infected by oral gavage with 10^8^ cells of the indicated strain. After 3 days, RNA was extracted from luminal contents of large intestines. The expression levels of indicated genes were determined by qRT-PCR analysis and shown as the means ± SDs. *n* = 3 biologically independent samples. Significance was determined using the unpaired two-tailed Student’s *t*-test. (**D**) GlcNAc fails to induce hypha-associated transcription in clinical isolates. Overnight cultures of SC5314 and clinical isolates were diluted at 1:100-fold into SC galactose medium and incubated at 30°C for 4 h. Log-phase cells were then treated with 50 mM GlcNAc at 37°C. Samples were collected at 3.5 h upon GlcNAc treatment for qRT-PCR analysis. Data are presented as the mean ± SD. *n* = 3 biologically independent samples. Significance was measured with the unpaired two-tailed *t*-test in GraphPad Prism. (**E and F**) Serum, but not GlcNAc, can induce elongated hyphae formation in clinical isolates of *C. albicans*. Overnight cultures of SC5314 and clinical isolates were diluted at 1:100-fold into SC galactose medium at 30°C and incubated for 4 h. Cells were then treated with 50 mM GlcNAc or 10% serum for hyphal induction. Photographs were taken after 3.5 h of incubation at 37°C. Representative images of three biologically independent experiments are shown. Scale bar, 10 µm. **P < 0.01, ***P < 0.001, ****P < 0.0001.

In microorganisms, evolutionarily conserved mechanisms facilitate adaptation to their new niches. To study mechanisms that underlie adaptive evolution in *C. albicans*, we analyzed 11 clinical isolates of *C. albicans* from patients who suffered from superficial candidiasis. To characterize the genetic background of these isolates, we carried out multilocus sequence typing analysis based on the sequences of seven housekeeping genes (21). We determined the distance between every two isolates using unweighted pair group method with arithmetic mean (UPGMA) to construct a phylogenetic tree (Fig. 1A), which infers the genetic diversity of these 11 clinical isolates of *C. albicans*.

Since routine laboratory media and culture conditions were totally different from conditions in natural niches, we speculate that clinical isolates of *C. albicans* would exhibit an advantage under *in vivo* conditions in comparison to the lab reference strain SC5314. To test this, a competition experiment was conducted in which mice were orally inoculated with 1:1 mixtures of a nourseothricin-resistant SC5314 and an unmarked strain of clinical isolates. The NAT marker was shown to have no effect on commensal fitness (22). Mice were treated with antibiotic water (streptomycin, 2 mg/mL; penicillin, 0.97 mg/mL) for 3 days prior to inoculation and throughout the commensal competition experiment to remove bacterial species. As expected, all 11 clinical isolates we tested are hypercompetitive in the gut colonization model such that they outcompeted the SC5314 strain at time points as early as 5 days after inoculation (Fig. 1B). This result reveals that SC5314 displays less fitness than clinical isolates when colonizing the gut, which is consistent with a previous study (23).

The hypha-associated transcription was shown to inhibit commensal fitness (18). We therefore examined the expression of hypha-associated genes including *UME6*, *SAP6*, and *HYR1*, all of which are involved in commensal fitness (18), in SC5314 and clinical isolates. As expected, we detected a dramatic increase in the expression of all three genes in SC5314 compared to that in N267429 and H71716 (Fig. 1C), two clinical isolates picked up randomly, during gut colonization. Our recent study suggested that GlcNAc serves as a major inducer for the hyphal transcriptional program in *C. albicans* in murine gut (24). We reasoned that clinical isolates of *C. albicans* form fewer filaments in response to GlcNAc, then exhibit enhanced competitive fitness during gut colonization. Consistent with this prediction, a much lower expression of hypha-associated genes was observed in clinical isolates upon GlcNAc induction than that in SC5314 (Fig. 1D). Correspondingly, GlcNAc failed to induce elongated filaments in clinical isolates, whereas most SC5314 cells formed elongated hyphae under the same condition (Fig. 1E). In contrast to GlcNAc, all 11 clinical isolates of *C. albicans* retained a normal filamentation pattern in response to serum (Fig. 1F). In support of this result, clinical isolates exhibited similar lethality to SC5314 in the murine model of systemic infection (Fig. S1). These data indicate that the defect in the hypha-associated program in clinical isolates is condition specific. Such morphology pattern might confer an advantage to clinical isolates in commensal growth but retains disease-causing potential in systemic infection.

### *In vitro* passaging enhances GlcNAc-responsive hyphae that confers commensal fitness disadvantage

**Fig 2:**
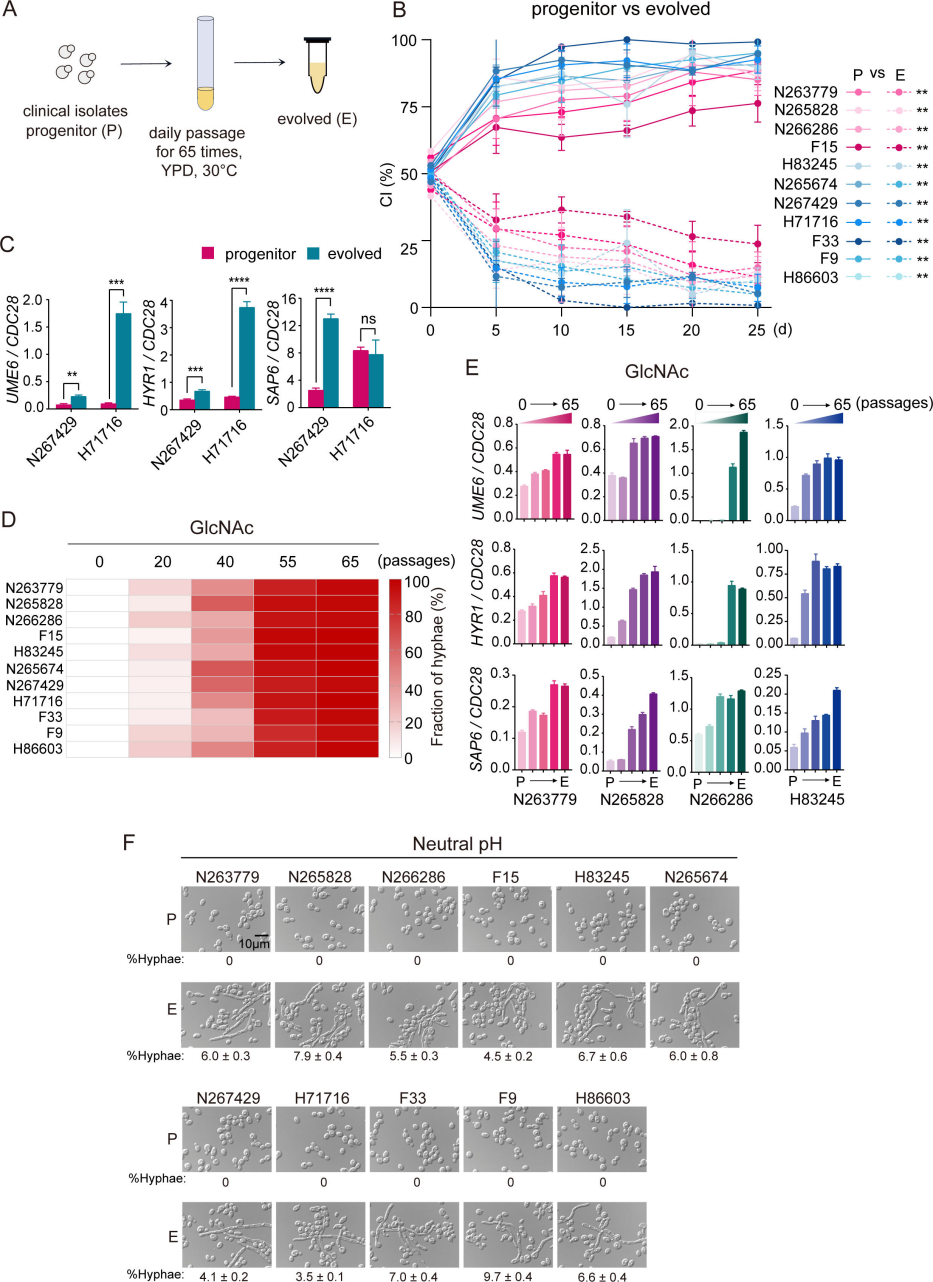
*In vitro* evolved strains exhibit a reduced commensal fitness relative to progenitors. (**A**) Schematic of *in vitro* passaging experiments. *C. albicans* cells were incubated in liquid YPD medium at 30°C for 24 h and then diluted at 1:100 into fresh YPD medium. After an additional incubation for 24 h, this procedure was repeated, and serial passaging was performed daily for a total of 65 passages. (**B**) The commensal fitness in competition between clinical isolates (P) and their *in vitro* derivatives (E) was examined as described in Fig. 1B. (**C**) Expression levels of *UME6*, *SAP6*, and *HYR1* in progenitor and *in vitro* evolved cells during commensal growth, determined as described for Fig. 1C. Data are presented as the mean ± SD of three independent experiments. Significance was determined using the unpaired two-tailed Student’s *t*-test. (**D and E**) GlcNAc-responsive hyphae formation (**D**) and hypha-associated transcription (**E**) are gradually recovered during *in vitro* passaging of clinical isolates. Heatmap (**D**) showing the ratio of hyphae of evolved cells in response to GlcNAc. Source data of panel **D** are shown in Fig. S2A. Hyphal induction in GlcNAc was performed as described in Fig. 1D. (**F**) Evolved cells fail to form elongated hyphae in neutral pH. Overnight cultures of progenitor and *in vitro* evolved cells were diluted at 1:100-fold into SC galactose medium at 30°C and incubated for 4 h. Cells were then treated with neutral pH for hyphal induction. The medium was buffered to pH 7 with HEPES. Photographs were taken after 3.5 h of incubation at 37°C. Representative images of three biologically independent experiments are shown. Scale bar, 10 µm. **P < 0.01, ***P < 0.001, ****P < 0.0001. ns, no significance.

The lab reference strain SC5314, which was originally isolated from a patient of candidemia (25, 26), has been grown and passaged under lab conditions for a long period. We reasoned that multiple *in vitro* passages shape the evolution of SC5314, leading to the reduced *in vivo* fitness. To test whether this is common to clinical isolates of *C. albicans*, *in vitro* passaging of each of the 11 isolates was performed daily under standard laboratory conditions (yeast extract peptone dextrose [YPD] medium, 30°C), and isolates were collected at various time points as long as 65 days (Fig. 2A). After 65 daily serial passages, fungal cells were harvested and were then orally inoculated with their corresponding progenitors at a 1:1 ratio to examine competitive fitness. As expected, evolved cells across all 11 lineages were outcompeted by their related progenitors in a direct competition experiment (Fig. 2B). A significant increase in hypha-associated transcription was detected in *in vitro* evolved cells during gut colonization compared to that of their related progenitors (Fig. 2C), which could contribute to the reduced commensal fitness. We then showed that all clinical isolates gradually gained capacity to form hyphae in response to GlcNAc after *in vitro* evolution (Fig. 2D; Fig. S2A). Next, four evolved lineages were randomly picked up for gene expression assay. As shown in Fig. 2E, the expression of hypha-associated genes was gradually increased upon GlcNAc induction, along with *in vitro* passaging. To confirm the effect of *in vitro* evolution on GlcNAc responsiveness of clinical isolates, all of the 11 clinical isolates were passaged in YPD plates at 30°C (Fig. S2B). One hundred sequential passages were performed in which cells were collected from YPD plates after 3 days of incubation and plated onto new YPD plates. As shown in Fig. S2B, cells that evolved in YPD plates also responded to GlcNAc to form filaments. It seems likely that this type of evolution specifically impacts GlcNAc-induced hyphae formation, as few evolved cells formed elongated hyphae in a medium with neutral pH (Fig. 2F). These results suggest that *in vitro* evolution attenuates commensal fitness in clinical isolates of *C. albicans* through promoting GlcNAc-responsive hypha-associated transcription.

### TOR pathway activity is suppressed in *in vitro* evolved cells

**Fig 3:**
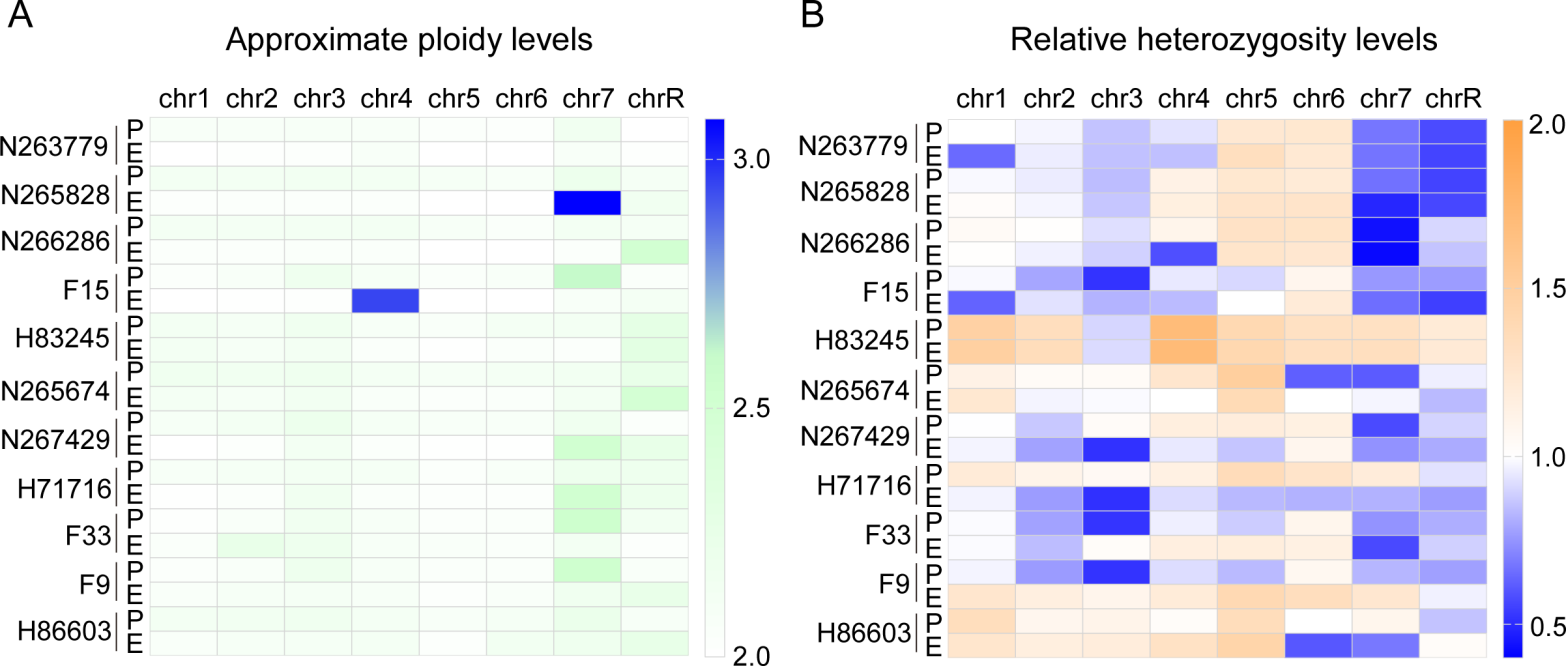
Genomic analysis of progenitor (P) and *in vitro* evolved (E) strains. (**A**) Heatmaps indicate estimated chromosome copy numbers across the eight *C. albicans* chromosomes based on coverage levels and normalized to whole-genome depth. (**B**) Relative heterozygosity levels of the indicated strains, normalized to SC5314.

To understand the mechanisms of how *in vitro* passaging drives the evolution of the GlcNAc-responsive cellular program in clinical isolates of *C. albicans*, we undertook whole-genome sequencing on 11 pairs of progenitor-evolved isolates. Because LOH (24, 27), copy-number variation (CNV) (28), and single-nucleotide polymorphisms (SNPs) contribute to the adaptive evolution of *C. albicans* (29, 30), we sought to identify potentially adaptive genetic changes between isolates within each pair by focusing on large-scale events (LOH and aneuploidies) as well as SNPs. Two previous studies passaged *C. albicans* in a murine model of GI commensalism and observed that isolates repeatedly acquired a trisomy of Chr 7 during passaging, which is associated with enhanced GI fitness (11). As shown in Fig. 3A, aneuploidy was observed in 1 of 11 evolved isolates involved in Chr 7 trisomies. Nevertheless, this observation could not explain the reduced competitive fitness of evolved strains, as the acquisition of an extra copy of chromosome 7 was shown to promote colonization. As previously reported, a wide variety of LOH events have been observed in *C. albicans* (14, 31). They did not, however, show any obvious trends during *in vitro* passaging (Fig. 3B). In addition, we could not detect any protein genetic polymorphisms in the *NGS1* or *REP1* gene in our genome sequencing analyses after *in vitro* evolution (Table S1). So far, *NGS1* and *REP1* are the only two genes known to be specifically required for GlcNAc-responsive hyphal growth (32, 33). These results underscore the need to investigate alternative mechanisms that enable *C. albicans* to undergo adaptive evolution.

**Fig 4:**
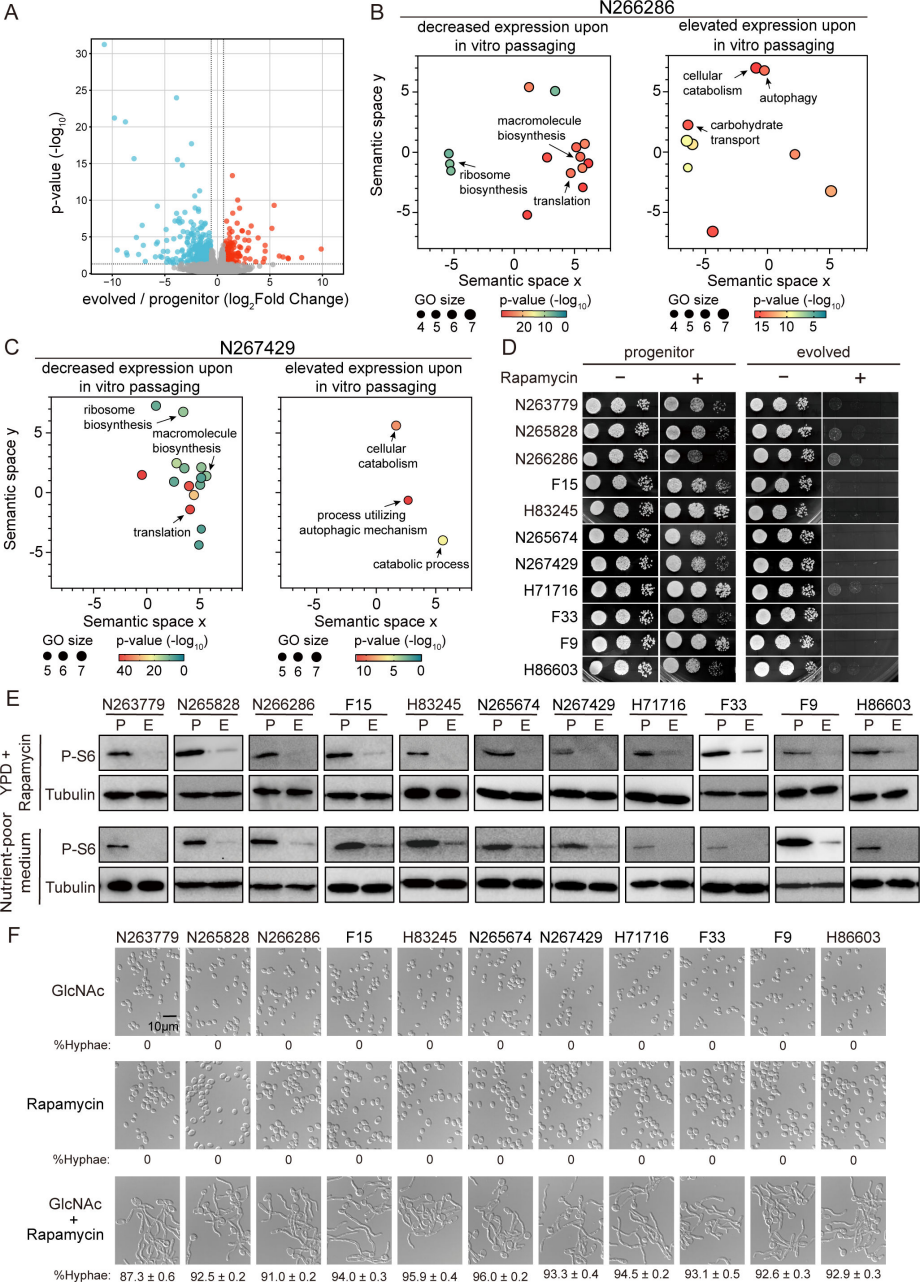
*In vitro* evolution leads to a reduction in TOR signaling. (**A**) Volcano plot of genes identified by RNA-seq of *in vitro* evolved cells compared to progenitor cells of N266286 in the gut colonization model. (**B and C**) GO terms for the differentially expressed genes (Benjamini–Hochberg) are represented in semantic similarity scatterplots for genes with decreased expression (left) or elevated expression (right) in *in vitro* evolved cells compared to progenitor cells from N266286 (**B**) and N267429 (**C**) isolates in the gut colonization model. (**D**) *In vitro* passaging confers hypersensitivity to rapamycin. Ten-fold serial dilutions from overnight cultures of progenitor and evolved cells from indicated isolates were spotted on solid YPD medium with or without 5 nM rapamycin at 30°C for 48 h before images were acquired. Images are representative of three biological replicates for each condition. (**E**) Overnight culture of progenitor and evolved cells of each isolate was diluted at 1:100 into YPD with 5 nM rapamycin or nutrient-poor medium (0.85 g/L YNB, 0.02 g/L L-tryptophan, 0.1% glucose). Cells were harvested after 6 h of incubation at 30°C. Western analysis was carried out using an anti-P-S6 antibody to indicate levels of TOR and with an antitubulin antibody for loading control. Representative blots of three independent experiments are shown. (**F**) Rapamycin addition enables clinical isolates of *C. albicans* to form elongated hyphae in GlcNAc. Overnight cultures of indicated clinical isolates were diluted at 1:100-fold into SC galactose medium at 30°C and incubated for 4 h. Cells were then treated with 50 mM GlcNAc, 5 nM rapamycin, or 50 mM GlcNAc combined with 5 nM rapamycin at 37°C for 3.5 h before images were acquired. Images are representative of three biological replicates for each condition.

To identify genetic pathways mediating adaptive evolution in *C. albicans*, two isolates, N266286 and N267429, were randomly picked up, and transcriptome analysis was conducted to compare the transcriptional responses between commensally propagated progenitor and evolved strains from each isolate during gut colonization. Animals were colonized with the progenitor or evolved strain (*n* = 3 mice/strain) for 3 days, and RNA was recovered from the large intestine. Using a 1.5-fold cutoff, we found that there are 582 genes downregulated and 362 genes upregulated in the evolved strain compared to the progenitor strain of N266286 in the gut (Fig. 4A). Gene Ontology (GO) term analysis (false discovery rate <0.05, Benjamini–Hochberg) indicates that the downregulated genes are enriched for translation, ribosome biosynthesis, and macromolecule biosynthesis, and the upregulated genes were enriched for carbohydrate transport, cellular catabolism, and autophagy (Fig. 4B), suggesting the repression of the TOR pathway in the evolved strain of N266286. We interrogated this phenomenon further using another isolate, N267429. The transcriptome analysis consistently showed a higher TOR activity in the progenitor strain and a lower TOR activity in the strain after *in vitro* passaging of N267429 (Fig. 4C). These transcriptome data suggest that *in vitro* evolution leads to a reduction in TOR activity in clinical isolates of *C. albicans*.

To ascertain whether changes in TOR activity occur during *in vitro* evolution across a broader range of *C. albicans* isolates, we compared the antifungal activity of the TOR inhibitor rapamycin in progenitor and evolved strains. For all 11 isolates, rapamycin was more potent in evolved strains relative to progenitor strains at 30°C (Fig. 4D). We next used a well-established assay to monitor TOR activity in *C. albicans* by measuring cellular levels of phosphorylated ribosomal protein S6 (P-S6) (34). As shown in Fig. 4E, all these 11 clinical isolates showed an observable P-S6 even in YPD upon rapamycin treatment, as well as when grown at a nutrient-poor medium (0.85 g/L YNB, 0.02 g/L L-tryptophan, and 0.1% glucose) at 30°C. In contrast, this phosphorylation in evolved cells that were passaged *in vitro* was almost undetectable under both conditions (Fig. 4E). The reduction in rapamycin tolerance and P-S6 in *in vitro* evolved cells could be also observed at 37°C (Fig. S2A and B), excluding the possibility that the lower TOR activity is a sign of “less stress” in cells evolved *in vitro* at 30°C. Together, the data revealed a molecular link between *in vitro* evolution and TOR signaling.

To explore the genetic basis for the TOR suppression in the evolved clinical isolates, the sequences of *TORC1*, as well as *BRG1* and *HOG1*, which are implicated in TOR signaling-mediated sustained hyphal development (35, 36), were checked through our whole-genome sequencing data. Although we identified a number of single-nucleotide variants in these three genes among the evolved strains, most of them were synonymous (Table S1). Moreover, the predicted protein variants identified across these three genes among the evolved strains did not show any obvious trends (Table S1). Because TOR activity and GlcNAc-responsive filamentation are negatively correlated in all *C. albicans* isolates, we speculated that TOR regulates GlcNAc responsiveness. In support of this conjecture, inhibition of TOR with rapamycin restored GlcNAc-induced filamentation in all 11 clinical isolates to levels comparable to those in strains after *in vitro* evolution, but rapamycin alone was unable to induce hyphae in the absence of GlcNAc (Fig. 4F). Adding rapamycin into the medium with neutral pH could not enhance filamentation either (Fig. S4), suggesting that rapamycin induces hyphae formation specifically in the presence of GlcNAc in clinical isolates.

To further characterize this phenomenon, four clinical isolates were passaged in YNB-based medium with glucose or GlcNAc as a carbon source to measure the impact of lab culture conditions on *in vitro* evolution of *C. albicans* (Fig. S5A). Interestingly, *in vitro* passaging in YNB with glucose or GlcNAc similarly decreased Tor1 activity (Fig. S5B) while promoting GlcNAc-responsive filamentation in evolved cells (Fig. S5C) as that passaged in YPD. We also did the same *in vitro* passaging experiments in SC5314 and two clinical isolates from patients of candidemia. As expected, SC5314 exhibited hypersusceptibility to rapamycin, extremely low TOR activity in the presence of rapamycin, and robust filamentation in response to GlcNAc, and *in vitro* passaging could not further enhance these phenotypes (Fig. S5D through F), supporting the notion that SC5314 has already been adapted to a lab environment. In contrast, a reduction in TOR activity and an increase in GlcNAc-responsive filamentation were observed in clinical isolates of candidemia after *in vitro* evolution (Fig. S5D through F). Collectively, these data reveal that the reduction in TOR activity due to *in vitro* evolution could promote increased GlcNAc-responsive hypha-associated transcription in *C. albicans*, thus contributing to a reduced fitness in the murine GI tract. This phenomenon is common to all the *C. albicans* clinical isolates we tested and is independent of culture conditions during passaging.

### *In vitro* evolution leads to a reduction in fitness toward stress response and *in vivo* infection

**Fig 5:**
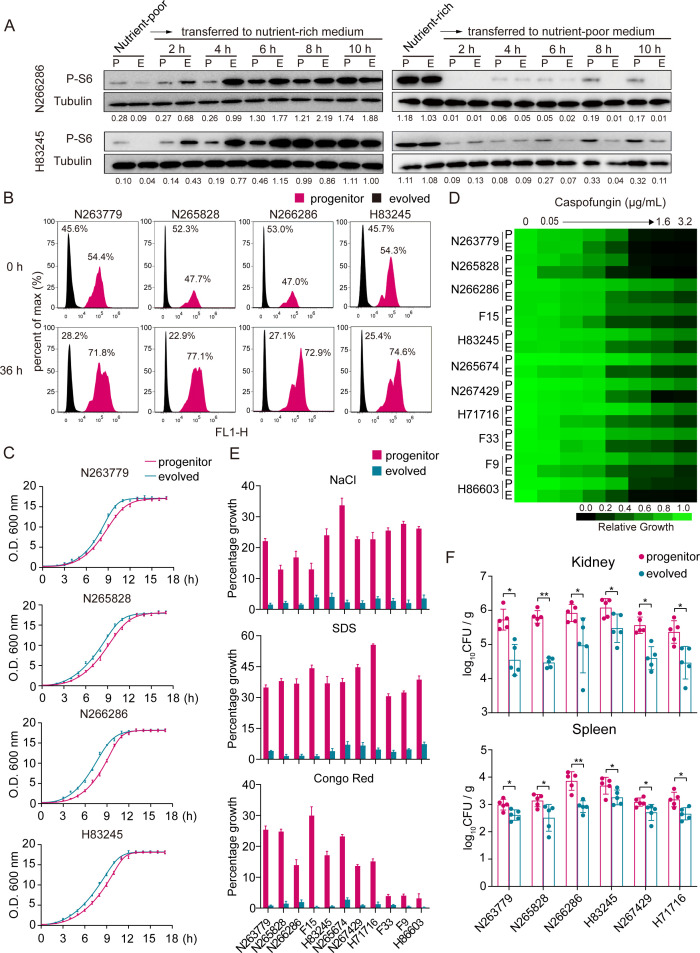
*In vitro* passaging confers a reduction in fitness toward stress response and systemic infection. (**A**) Western blotting showing the dynamics of TOR activity in progenitor and evolved cells. An overnight culture of progenitor and evolved cells, which was incubated in nutrient-poor medium (0.85 g/L YNB, 0.02 g/L L-tryptophan, 0.1% glucose), was diluted 1:50 into YPD (nutrient-rich medium) at 30°C. Conversely, progenitor and evolved cells were cultured overnight in YPD and were then diluted 1:50 into nutrient-poor medium at 30°C. Cells were then collected at indicated time points, and the cell lysate was prepared for Western blotting. Representative blots of three independent experiments are shown. (**B**) *In vitro* evolved cells display a reduced fitness relative to progenitor cells upon starvation. Progenitor cells carrying Hsp90-GFP and *in vitro* evolved cells were mixed at a 1:1 ratio and were then incubated in nutrient-poor medium (0.85 g/L YNB, 0.02 g/L L-tryptophan, and 0.1% glucose) for 36 h at 30°C. The resulting cells were subjected to flow cytometry analysis detecting Hsp90-GFP fluorescence. Representative results of three independent experiments are shown. (**C**) *In vitro* evolution confers a fitness benefit in nutrient-rich medium. The change in optical density over time as a measure of growth was monitored in liquid YPD of progenitor and evolved cells at 30°C. *n*  =  3 biologically independent samples. (**D**) *In vitro* evolved cells are hypersensitive to caspofungin. Caspofungin susceptibility assays were conducted in YPD medium. Growth was measured by absorbance at 600 nm after 48 h at 30°C. Optical densities were averaged from three measurements. Data are quantitatively displayed in heatmap format (see color bar). (**E**) About 200 progenitor or *in vitro* evolved cells were plated on YPD plates or YPD plates containing 1.5 M NaCl, 0.25% SDS, or 200 µg/mL Congo red, and they were incubated at 30°C for 72 h. The percentage of growth is calculated as a ratio of the colonies formed in drug-containing plates versus YPD plates. Results are shown as mean ± SD of three independent experiments. (**F**) *In vitro* passaging results in a reduction in fitness during *in vivo* infection. Groups of male BALB/c mice were infected with 5 × 10^5^ CFU of progenitor or *in vitro* evolved cells of indicated clinical isolates, followed by euthanasia of three animals per group after 5 days. CFUs were determined by plating kidney or spleen homogenates onto Sabouraud agar (supplemented with streptomycin and ampicillin) and counting after 2 days. Data represent the mean and standard deviations of three independent experiments. *P < 0.05, **P < 0.01.

To further characterize the differences in kinetics of the TOR pathway between clinical isolates and their *in vitro* passaged derivatives, a time course analysis of TOR activity, which is indicated by S6 phosphorylation levels, was performed during transition from a nutrient-poor medium to YPD (nutrient-rich medium) and vice versa. As described earlier, the progenitor cells of both clinical isolates we tested displayed a higher TOR activity relative to *in vitro* evolved strains under nutrient-poor conditions (Fig. 5A). When they were transferred to YPD, an elevated TOR activity was detected in evolved strains as fast as 2 h of incubation and remained high over the time course, whereas a dramatically slower increase in the dynamics of TOR activity was observed in progenitor cells under the same conditions (Fig. 5A). Conversely, the progenitor and evolved strains exhibited comparable high TOR activity in YPD, which was decreased sharply after 2 h incubation in the nutrient-poor medium in both strains (Fig. 5A). Interestingly, P-S6 levels revealed a restoration of TOR activity in progenitor strains at 6 h of incubation in the nutrient-poor medium, which remained for at least 10 h (Fig. 5A), although the levels were much lower than that in YPD. However, the S6 phosphorylation in evolved strains was consistently low over the time course in the nutrient-poor medium. These data support a model in which *in vitro* evolution leads to a reduction in the basal level of TOR activity but confers a hypersensitivity to extracellular nutrient conditions. Indeed, clinical isolates displayed a significant fitness advantage relative to their *in vitro* passaged derivatives when grown in nutrient-poor medium at both 30°C and 37°C (Fig. 5; Fig. S6A). This was accompanied by a decrease in fitness in rich medium as saturated cells of clinical isolates exhibited a delay in reaching log-phase growth state when they were released into YPD (Fig. S5C and S6B).

To explore the possibility that the enhanced TOR activity reflects adaptation to other factors besides commensal fitness, we measured phenotypes associated with pathogenicity, including antifungal drug tolerance, stress response, and *in vivo* fitness during invasive infection in progenitor cells and their *in vitro* passaged derivatives. The drug sensitivity assay showed that, although clinical isolates and *in vitro* evolved strains exhibited comparable fluconazole susceptibility (Fig. S7), the latter were more sensitive to caspofungin (Fig. 5D). In addition, evolved cells were markedly more sensitive to NaCl than progenitor cells; evolved cells were also more sensitive to the membrane stress caused by sodium dodecyl sulfate (SDS) and the cell wall stress caused by Congo red (Fig. 5E). Finally, we determined whether *in vitro* evolution impacts *in vivo* fitness during systemic infection. As shown in Fig. 5F, infection with *in vitro* evolved *C. albicans* cells displayed a reduced fungal burden in both kidneys and spleens relative to that infected with cells of clinical isolates. Taken together, our data reveal that *in vitro* evolution confers an increased susceptibility to multiple stresses and a reduction in fitness during *in vivo* commensal growth and systemic infection.

### The fitness advantage in clinical isolates relies on the TOR pathway

**Fig 6:**
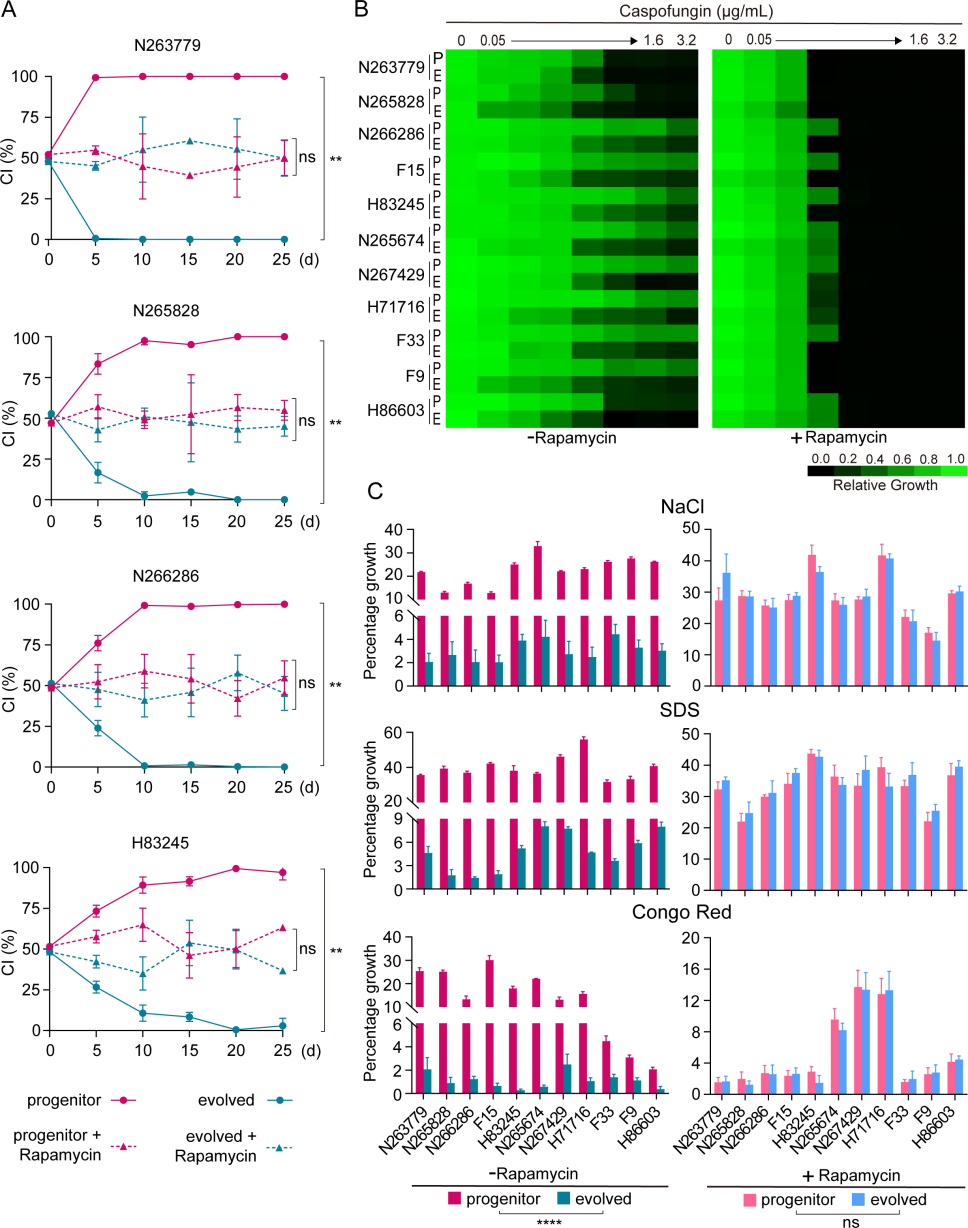
Pharmacologic inhibition of TOR by rapamycin suppresses the fitness advantage in clinical isolates. (**A**) The commensal fitness of *in vitro* evolved cells and progenitor cells in mice treated with rapamycin (dashed line) or vehicle (solid line) was determined as in Fig. 1B. (**B**) Progenitor and *in vitro* evolved cells were cultured in YPD containing different concentrations of caspofungin with or without 5 nM rapamycin. Growth was measured by absorbance at 600 nm after 48 h at 30°C. Data represent the means from three independent experiments. (**C**) Progenitor or *in vitro* evolved cells were plated on YPD plates and YPD plates containing stress-causing agents in the presence or absence of 5 nM rapamycin and incubated at 30°C for 72 h. The percentage of growth is calculated as a ratio of the colonies formed in drug-containing plates with or without rapamycin versus YPD plates with or without rapamycin, respectively. Results are shown as mean ± SD of three independent experiments. **P < 0.01, ****P < 0.0001. ns, no significance.

We reasoned that if high basal levels of TOR activity confer a fitness benefit toward stresses, then the fitness advantage could be suppressed by experimental inhibition of TOR signaling in clinical isolates of *C. albicans*. To test this hypothesis, cells of clinical isolates and their *in vitro* passaged derivatives were orally inoculated with 1:1 mixtures in mice fed with rapamycin or vehicle only to examine competitive fitness. As described earlier, evolved cells exhibited diminished fitness compared with progenitor cells across all four isolates we tested in control mice. By contrast, no significant difference in commensal fitness was observed in mice fed with rapamycin (Fig. 6A). Similarly, clinical isolates and their *in vitro* passaged derivatives exhibited no significant difference in susceptibility to caspofungin, NaCl, Congo red, or SDS upon treatment with rapamycin (Fig. 6B and C). These data indicate that inhibiting TOR signaling is sufficient to abrogate the fitness advantage in clinical isolates. Thus, we suggest that *C. albicans* undergoes adaptive evolution via modulating TOR activity.

## DISCUSSION

*C. albicans* is the primary fungus of the human gut microbiota but remains a perpetual threat with the ability to infect multiple niches, especially in immunocompromised hosts. The fungus lives and coevolves in these complex environments of the human body. Previous studies have focused on genome evolution as a driver of adaptive processes. Here, we demonstrate that *C. albicans* undergoes adaptive evolution via modulation of TOR signaling. Our data reveal that changes in TOR activity underlie some evolved traits, with important consequences for both host adaptation and pathogenicity.

*C. albicans* cells usually have two genetically distinct copies of every gene and lack a complete sexual cycle (37). Point mutations such as base substitutions are an important driver of genomic change due to their high frequency of production and likelihood of being tolerated (13, 38, 39). For example, passaging of *C. albicans* lab reference strain SC5314 in the murine gut revealed selection for mutations of *FLO8*, a transcriptional regulator that promotes filamentation (40). Similarly, loss-of-function mutations in *EFG1*, another key regulator of filamentation (41), were often observed during GI passage of clinical isolates (42). Either *EFG1* or *FLO8* deletion mutants outcompeted wild-type strains when directly evaluated in the GI (40, 43) yet showed reduced virulence in the model of systemic infection (16, 44), indicating that the fitness advantage in commensal growth due to inability to form hyphae is counterbalanced by a virulence penalty. This raises a question of how *C. albicans* commensalism has evolved with retention of virulence traits. In this study, we showed that all clinical isolates we tested exhibited increased commensal fitness in competition with the lab reference strain SC5314 but retained comparable disease-causing potential (Fig. 1; Fig. S1). *In vitro* passaging caused the reduced TOR activity in evolved cells (Fig. 4E), leading to attenuated fitness when colonizing the gut and during systemic infection (Fig. 2B and 5F), as well as in stress response (Fig. 5D and E), relative to progenitor cells. This phenomenon is consistent across *in vitro* evolved isolates and is independent of genetic background and *in vitro* passaging environment. Thus, it can be argued that clinical isolates of *C. albicans* gain an elevated TOR activity when living in host niches, which confers an enhanced fitness during host adaptation.

Our previous work has demonstrated that GlcNAc, an abundant carbon source in the GI tract, represents a major inducer for the expression of hypha-associated genes, which dictates the commensal fitness of *C. albicans* (20). Supporting this notion, clinical isolates failed to express hypha-associated genes in GlcNAc and during gut colonization (Fig. 1C and D) but exhibited normal filamentation in response to serum (Fig. 1F). As a result, they are hypercompetitive during commensal growth but at the same time retain comparable virulence as the lab reference strain SC5314 in systemic infection. Furthermore, inhibition of TOR by rapamycin in clinical isolates led to increased GlcNAc-responsive hyphae formation (Fig. 4F). Thus, our work links the TOR pathway to GlcNAc signaling in *C. albicans*. Although GlcNAc is used by this fungus as a signal for nutrient availability (45), which is linked to TOR, whether and how TOR regulates GlcNAc-responsive cellular programs needs to be further investigated.

The TOR signaling pathway is widely expressed in eukaryotic cells and functions as an evolutionarily conserved sensor of environmental and endogenous stress (46–48). This confers an advantage of TOR in driving evolution as changes in TOR activity could affect many cellular processes without the need for generating genome variations. In support of this, inhibition of the TOR pathway with rapamycin is sufficient to abolish the fitness advantage of clinical isolates relative to their *in vitro* passaged derivatives (Fig. 6A through C). In addition, no changes were observed in either gene sequences or expression levels in *EFG1* and *FLO8* after *in vitro* passaging (see our genome sequencing and RNA-seq data). Thus, we suggest that modulation of TOR signaling serves as an efficient means of physiological adaptation of *C. albicans*. Increasing evidence has revealed a critical role of TOR-mediated cellular responses toward stress (49–52), but our study highlights the TOR signaling pathway directly driving adaptive evolution in a commensal-pathogenic fungus that must be proficient at adapting to cues to survive in the context of the complex host environment. Such a mechanism may be exploited by other organisms.

## MATERIALS AND METHODS

### Media and growth conditions

Clinical isolates of *C. albicans* were obtained from Shanghai Dermatology Hospital, Tongji University, Shanghai, China (Table S2). *C. albicans* strains were routinely grown at 30°C in YPD (2% Bacto Peptone, 2% glucose, 1% yeast extract). Transformants were selected on YPD plate supplemented with 200 µg/mL nourseothricin. YPD and YNB (0.17% Difco yeast nitrogen base without ammonium sulfate and 0.5% ammonium sulfate) with 2% glucose or GlcNAc were used for *in vitro* passaging.

Hyphal induction in liquid medium was conducted as described previously (32). Briefly, *C. albicans* cells grown overnight at 30°C in liquid YPD were washed three times with phosphate-buffered saline (PBS), resuspended in an equal volume of PBS, and diluted 1:100 into liquid SC medium containing 50 mM galactose. After 4 h of incubation at 30°C, hyphal growth was induced by a shift in temperature to 37°C in combination with 50 mM GlcNAc. In addition to GlcNAc, 10% serum and neutral pH were used for morphology assay in this study as well. For neutral pH condition, cells were pelleted and resuspended in synthetic complete (SC) galactose medium buffered to pH 7 with 15 mM HEPES buffer (pH 7). Cell morphology was detected using differential interference contrast optics at 3.5 h after transferring to 37°C.

### Plasmid and strain construction

The full length of green fluorescent protein (GFP) was introduced to the C-terminus of Hsp90 of clinical isolates using the CRISPR-Cas9 strategy as follows. The single-guide RNA (sgRNA) (5′-ATTTGAAGTTGATTAAACACCAGAAGG and 5′-AAAACCTTCTGGTGTTTAATCAACTTC) was annealed to insert into the pV1393 vector. The resulting plasmid was linearized by digestion with KpnI and SacI and transformed into clinical isolates. The GFP sequence was PCR amplified with the primers (5′-GACGAACCAGCTGGAGAATCTGCTATGGAAGAAGTTGATATGTCTAAAGGTGAAGAATT and 5′-AAAACTTATTTAACTAGAAAACTGTAGCCCTTCTGGTGTTTATTTGTACAATTCATCCA) from the plasmid pHL471 (53). The resulting PCR product was used as the repair template that was transformed into clinical isolates with sgRNA to produce Hsp90-GFP strains. The Hsp90-GFP fusion was verified by sequencing.

### Commensal competition experiments in mouse

All animals were singly or doubly housed, depending on the experiment, and provided autoclaved distilled water and autoclaved mouse chow. Female BALB/c mice (6–8 weeks old) (purchased from Beijing Vital River Laboratory Animal Technology Company) were treated with antibiotic water (streptomycin, 2 mg/mL; penicillin, 0.97 mg/mL) for 3 days and then inoculated with a 1:1 mixture of a nourseothricin-resistant strain and an unmarked strain at 5×10^8^ cells/mL by oral gavage as previously reported (22). The antibiotics water was used throughout the commensal competition experiment. Colonization was tested over time by collecting fresh fecal pellets and plating homogenates on YPD plates containing streptomycin (100 µg/mL) and ampicillin (50 µg/mL) supplemented with or without 200 μg/mL nourseothricin. The competitive index of the competition experiment has been shown as the proportion of the indicated strain to the total.

Rapamycin (1 mg/kg) was administered to mice by intraperitoneal injection once daily to inhibit TOR signaling. The same volume of the vehicle was served as control.

### Antifungal susceptibility testing

Susceptibility to fluconazole, caspofungin, or a combination of caspofungin and rapamycin was assayed in 96-well microtiter plates (Thermo, Waltham, MA, USA) as previously described (54). Assays were performed in a total volume of 0.1 mL/well with various concentrations of each drug in YPD medium. Plates were incubated in the dark at 30°C for 48 h before the optical density at 600 nm was determined using a spectrophotometer (BioTek Instruments).

### RNA sequencing and analysis

Female BALB/c mice (6–8 weeks old) were colonized with a single *C. albicans* strain (progenitor or *in vitro* evolved strain). Three animals were colonized with each strain for 3 days and housed individually. Animals were euthanized, and the content in the large intestine was flash frozen in liquid nitrogen and stored in −80°C. Library preparation and sequencing of RNA were performed at Novogene (Beijing, China). A sequencing library was constructed by using a NEBNext Ultra RNA library prep kit for Illumina (New England BioLabs, USA). The RNA-seq library was assessed by the Agilent Bioanalyzer 2100 system and quantified by quantitative reverse transcription PCR (qRT-PCR) before sequencing on the Illumina NovaSeq platform. Clean reads were mapped to the *C. albicans* reference genome (SC5314_A21), and mapped reads were counted. Differentially expressed genes were defined by a fold change of ≥1.5 and a *P* value of <0.05 obtained by DESeq2. GO term process analysis was performed on the Candida Genome Database (http://www.candidagenome.org/cgi-bin/GO/goTermFinder), and Benjamini–Hochberg adjustment for multiple comparisons was determined using DESeq2.

### RNA extraction and quantitative PCR expression analysis

Total RNA from *C. albicans* cells incubated under the *in vitro* conditions was purified using the RNAprep pure Tissue Kit and DNase-treated at room temperature for 15 min using the RNase-free DNase Set (Tiangen). Total RNA in the contents from the large intestines of mice was extracted using the Fecal RNA Extraction Kit (Biotech). cDNA was synthesized using the Maxima H Minus cDNA Synthesis Master Mix with dsDNase (Thermo), and qPCR was done using the iQ SYBR Green Supermix (Bio-Rad). The primers for qRT-PCR were described in our previous study (20). All data showed the average of three independent biological replicates with error bars representing the SD.

### Whole-genome sequencing of *C. albicans* strains

Genomic DNA was extracted using the plant genome DNA extraction kit (Sangon Biotech Co., Ltd., Shanghai, China). The library preparation and next-generation sequencing were performed by Sangon Biotech Co., Ltd. Quantified DNA (500 ng) was randomly fragmented by Covaris (Woburn, USA). Whole-genome sequencing libraries were prepared using Illumina Hieff NGS MaxUp II DNA Library Prep Kit (YEASEN, Shanghai, China). The library concentration and size were confirmed by Qubit (version 4.0) (Thermo) and 2% agarose gel electrophoresis, respectively. Then, the libraries were pooled and loaded on Novaseq 6000 (Illumina, San Diego, USA)/DNBseq-T7 (BGI, Shenzhen, China) to generate indexed paired-end reads of 2 × 150 bp.

Raw reads containing adaptor sequences and those with ambiguous or low-quality bases at the beginning or end were trimmed using Fastp. The qualified reads from each sample were aligned to the assembled *C. albicans* SC5314 reference genome (http://www.candidagenome.org/download/sequence/C_albicans_SC5314/Assembly21/current/) using BWA (version 0.7.17) with default parameters. Duplicated reads were removed, and coverage values were calculated using SAMTOOLS. Primary variation calling was conducted using the Genome Analysis ToolKit (GATK, v4.1.2). The bam file produced from the mapping procedure was analyzed for structural variation detection by DELLY (version 0.8.1) with default parameters. CNVs were detected with CALL in CNVnator (version 0.4). Functional annotation of all the genetic variants was completed by snpEff (version 4.3t).

### Ploidy and copy number variation

The following procedure was adapted and previously described in reference (11). To examine ploidy variation across the genome, the Illumina read alignment depth was calculated for 100 bp windows across the genome, using BEDTools (version 2.18) (55), SAMtools (version 1.3) (56), and the GATK (version 3.7) Depth of Coverage module. The read depth was calculated as the number of bases aligned per window divided by the length of the window and normalized to the average depth for each strain and to the GC content (57). The read depth was also normalized per the effective window length by removing any ambiguous sites in the respective window. The normalized alignment depth for each 100 bp window was then plotted, and large-scale variations in ploidy (twofold up or down coverage) were identified. These include whole chromosome and segmental aneuploidies larger than 0.1 Mbp. Smaller regions showing read depth variation were designated as CNVs, and their numbers were plotted based on the nature of the variation (twofold up or down coverage).

### LOH analysis

To identify LOH events, all variants were classified based on how each mutation alters heterozygosity at the respective site: LOHs, gains of heterozygosity, or mutations that do not alter heterozygosity (het neutral). LOH tracts were defined using each heterozygous site identified to have undergone LOH, and the size of the tracts was determined by visual inspection in IGV.

### Fluorescence-activated cell sorting

Fluorescence-activated cell sorting was conducted by using the Beckman CytoFLEX SRT system. The FL1-H channel was used for GFP detection, and a total of 10,000 cells in suspension were counted per sample. Results were analyzed with FlowJo cytometry analysis software.

### Murine model of systemic infection

BALB/c mice were purchased from Beijing Vital River Laboratory Animal Technology Company. Mice were housed in a temperature-constant animal room (22°C) with reversed dark/light cycle (7:00 a.m. on and 7:00 p.m. off) and 40%–70% humidity.

BALB/c mice were purchased from Beijing Vital River Laboratory Animal Technology Company. Mice were housed in a temperature-constant animal room (22°C) with reversed dark/light cycle (7:00 a.m. on and 7:00 p.m. off) and 40–70% humidity.

### Western blotting

Whole-cell lysate of *C. albicans* cells was prepared in a lysis buffer (50 mM Tris, pH 7.5, 100 mM NaCl, and 0.1% NP-40), supplemented with 1 mM phenylmethylsulfonyl fluoride (Biosharp) and one protease inhibitor cocktail tablet per 25 mL (Roche). The protein concentration of the cell lysate was accurately measured using the Bradford method. The lysate was then mixed with SDS-loading buffer and heated to 100°C for 10 min. The samples were separated by sodium dodecyl sulfate–polyacrylamide gel electrophoresis and transferred to a PVDF membrane. Subsequently, the membrane was probed with the appropriate antibodies for detection.

### Statistical analysis

All experiments were performed with at least three biological repeats except as indicated in the figure legends, and no statistical method was used to predetermine sample sizes. Analyses were conducted using GraphPad Prism (version 9.0) software. Results are expressed as the mean ± SD as indicated and analyzed using Student’s *t*-test. The surviving curve was analyzed using the log-rank test. *P* values of less than 0.05 were considered statistically significant.

## Data Availability

RNA-seq data and genome sequencing data that support the findings of this study have been deposited in the Genome Sequence Archive under the accession code CRA016765 and CRA016764, respectively. Reference genomes and genome annotations were obtained from the Candida Genome Database. The strains generated in this study are available from the corresponding author upon request.

## SUPPLEMENTAL MATERIAL

Supplemental figures mbio.03947-24-s0001.pdf Figures S1-S7.

Table S1 mbio.03947-24-s0002.xlsx SNVs in *in vivo*-evolved strains.

Table S2 mbio.03947-24-s0003.xlsx Clinical isolates used in this study.

ASM does not own the copyrights to Supplemental Material that may be linked to, or accessed through, an article. The authors have granted ASM a non-exclusive, world-wide license to publish the Supplemental Material files. Please contact the corresponding author directly for reuse.
