# Supplementary material for: Adaptive evolution of Candida albicans through modulating TOR signaling
Source: mBio. 2025 Mar 4;16(4):e03947-24. doi: 10.1128/mbio.03947-24 (PMC11980384; doi:10.1128/mbio.03947-24)
Supplement: Supplemental figures — Figures S1-S7. [file mbio.03947-24-s0001.pdf]

Supporting Information

Adaptive evolution of *Candida albicans* through modulating TOR signaling

Yaling Zhang[1], Lianjuan Yang[2], Youzhi Zhao[1], Kang Xiong[3], Hao Cui[1], Tianxu Wang[1], Xiaoping Liu[2], Chang Su[3], and Yang Lu[1*]

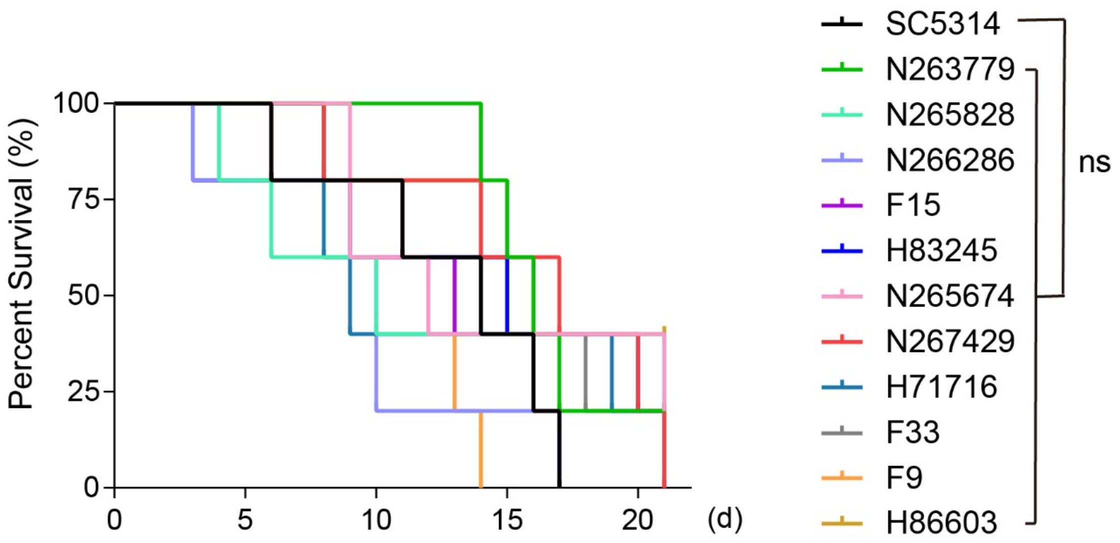

Fig. S1 Clinical isolates exhibited comparable virulence with SC5314 in systemic infection. 18-21 g ICR mice were inoculated with 4×10^5 cells of indicated strains by tail vein injection. n = 5 mice. Significance was measured with log-rank test. ns, no significance.

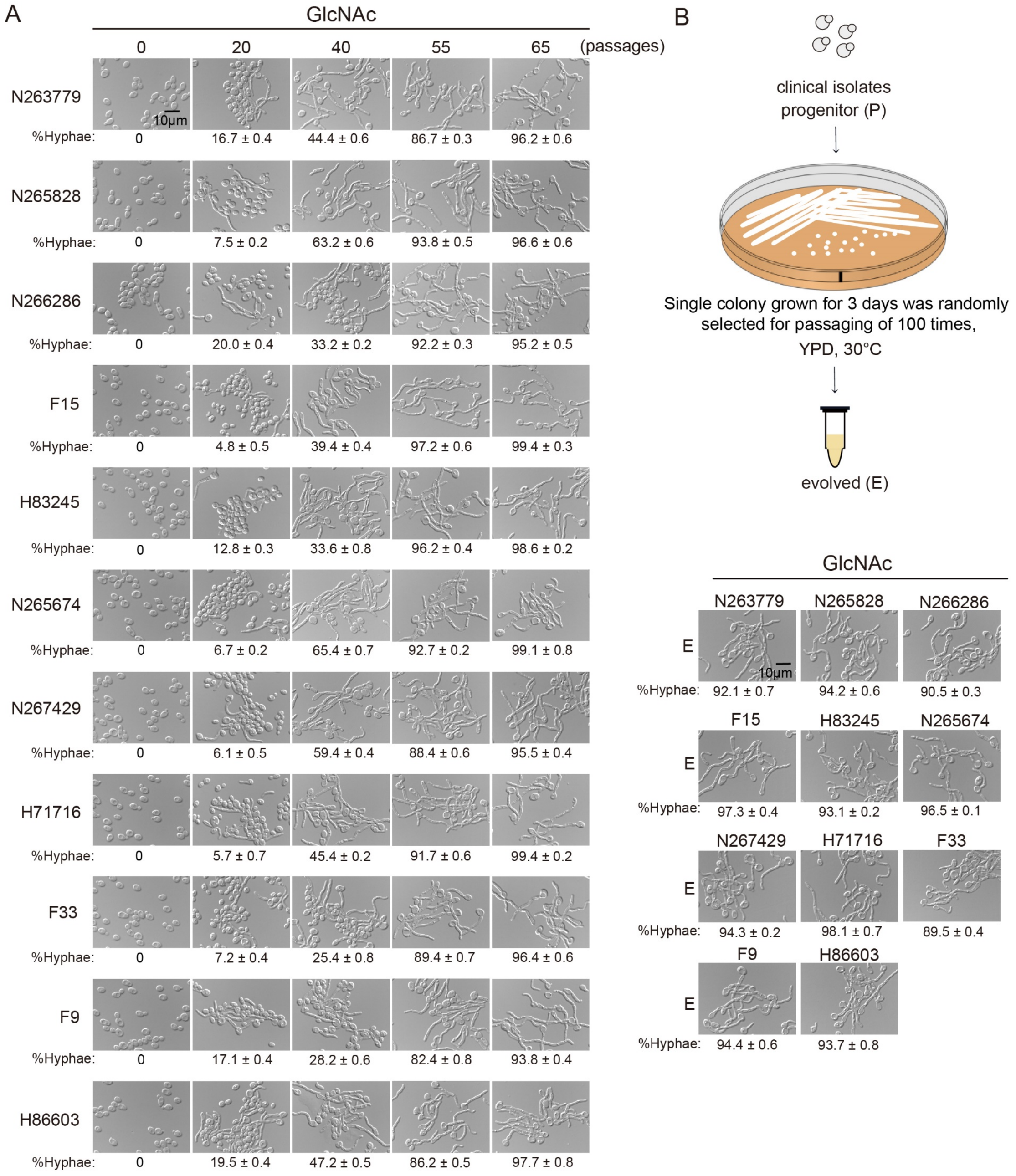

Fig. S2 In vitro passaging promotes hyphae formation in GlcNAc in evolved cells. (A) Cell morphology analysis for indicated strains during in vitro passaging. The percentage of hyphal cells was determined by counting at least 200 cells/sample. Cells which had a length-width ratio of >4.5 and characteristic shape were considered as hyphae. The data are represented as mean ± SD of three independent experiments. (B) In vitro passaging in YPD plates similarly promotes GlcNAc-responsive hyphae formation as that in liquid YPD medium.

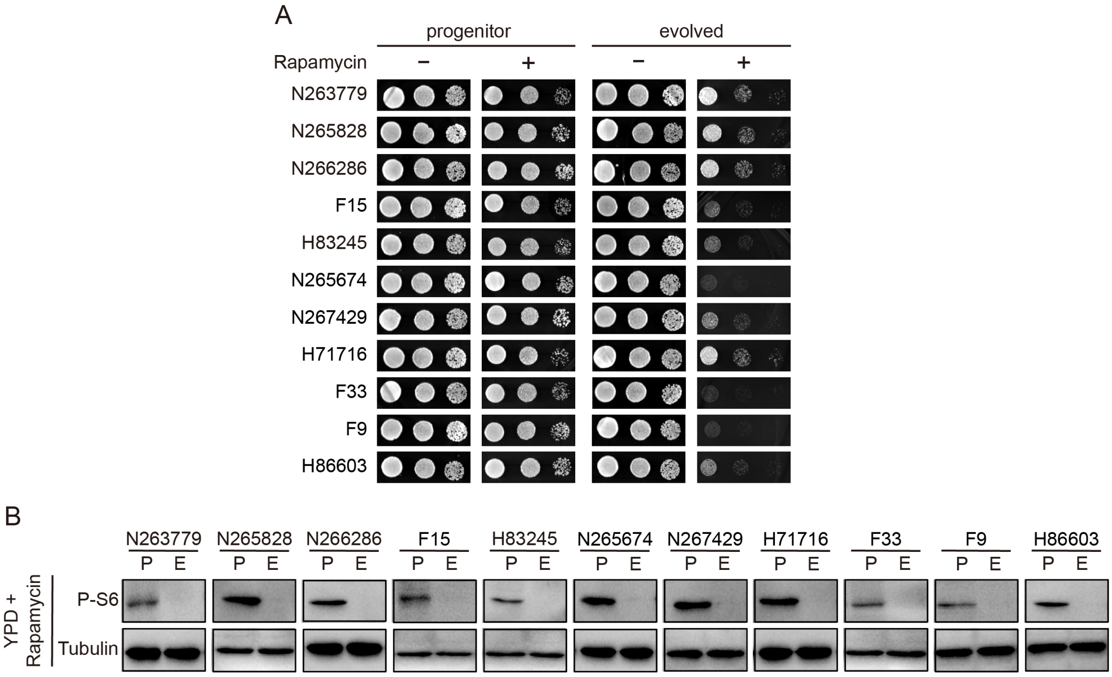

Fig. S3 *In vitro* passaging confers hypersensitivity to rapamycin and low TOR activity at 37°C. (A) Ten-fold serial dilutions from overnight cultures of progenitor and evolved cells from indicated isolates were spotted on solid YPD medium with or without 5 nM rapamycin at 37°C for 48 h before images were acquired. Images are representative of three biological replicates for each condition. (B) Overnight culture of progenitor and evolved cells of each isolate was diluted at 1:100 into YPD with 5 nM rapamycin at 37°C. Cells were harvest after 6 h of incubation. Western analysis was carried out using an anti-P-S6 antibody to indicate levels of TOR, and with an anti-tubulin antibody for loading control. Representative blots of three independent experiments are shown.

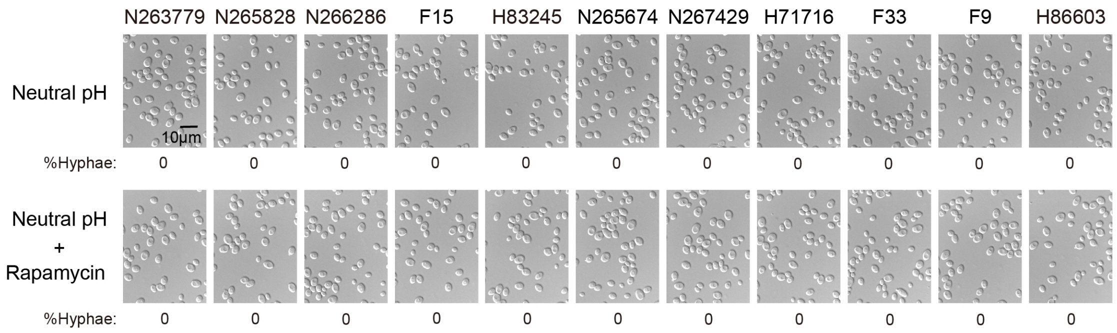

Fig. S4 Rapamycin does not induce filamentation in medium with neutral pH. Overnight cultures of indicated clinical isolates were diluted at 1:100 fold into SC galactose medium at 30°C and incubated for 4 h. Cells were then transferred into the medium buffered to pH 7 with HEPES in the presence or absence of 5 nM rapamycin. Photographs were taken after 3.5 h of incubation at 37°C. Representative images of three biologically independent experiments are shown. Scale bar, 10 μm.

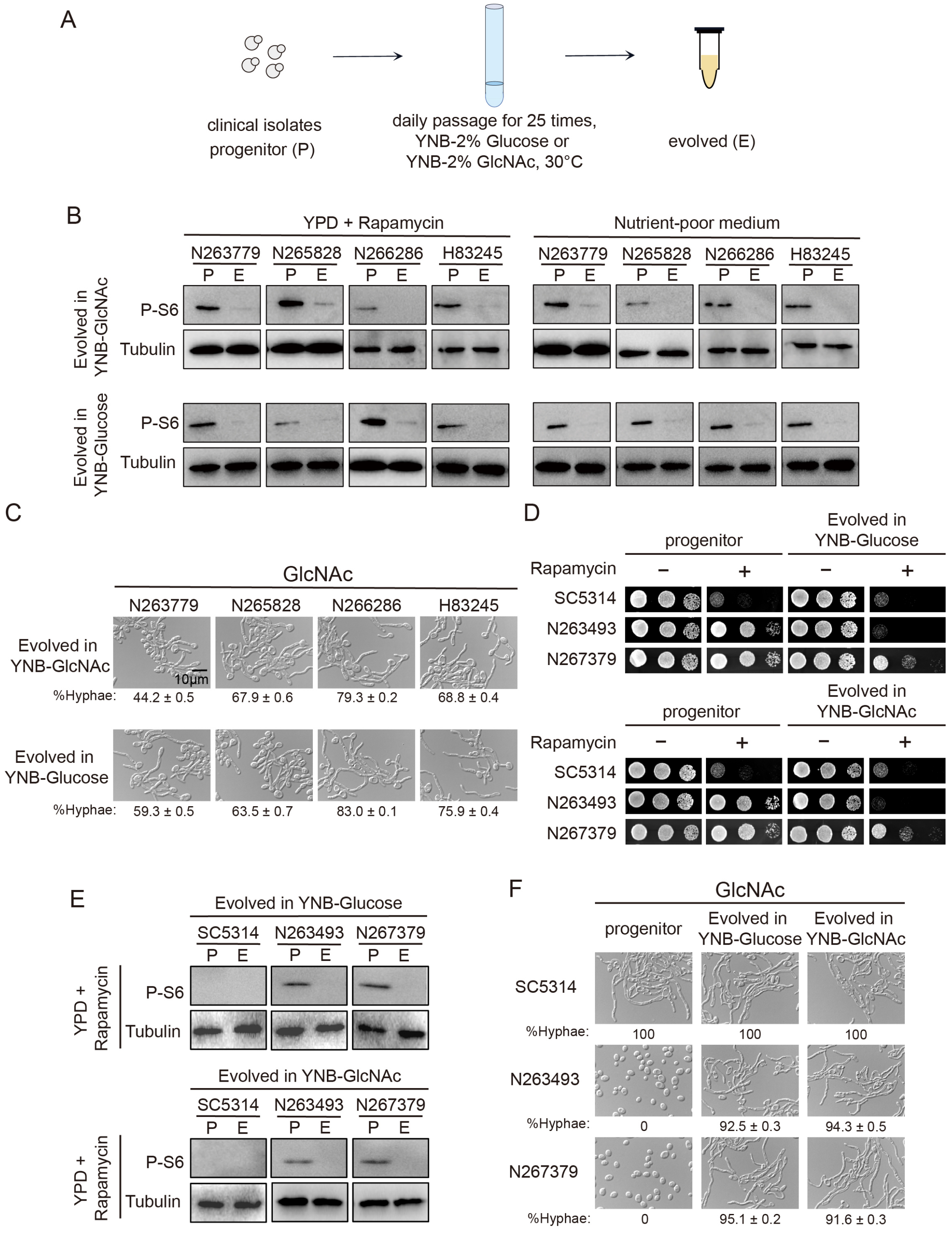

Fig. S5 The pattern of *in vitro* evolution in *C. albicans* is independent of environment. (A) Schematic of passaging in YNB with glucose or GlcNAc. (B) In vitro passaging in YNB with glucose or GlcNAc also promotes hyphae formation in response to GlcNAc. (C) Dephosphorylation of P-S6 after passaging in YNB with glucose or GlcNAc as that in YPD. (D) Ten-fold serial dilutions from overnight

cultures of progenitor and evolved cells from indicated isolates were spotted on solid YPD medium with or without 5 nM rapamycin at 30°C for 48 h before images were acquired. Images are representative of three biological replicates for each condition. (E) Overnight culture of progenitor and evolved cells of each isolate was diluted at 1:100 into YPD with 5 nM rapamycin. Cells were harvest after 6 h of incubation at 30°C. Western analysis was carried out using an anti-P-S6 antibody to indicate levels of TOR, and with an anti-tubulin antibody for loading control. Representative blots of three independent experiments are shown. (F) Overnight cultures of progenitor and in vitro evolved cells were diluted at 1:100 fold into SC galactose medium at 30°C and incubated for 4 h. Cells were then treated with 50 mM GlcNAc for hyphal induction. Photographs were taken after 3.5 h of incubation at 37°C. Representative images of three biologically independent experiments are shown.

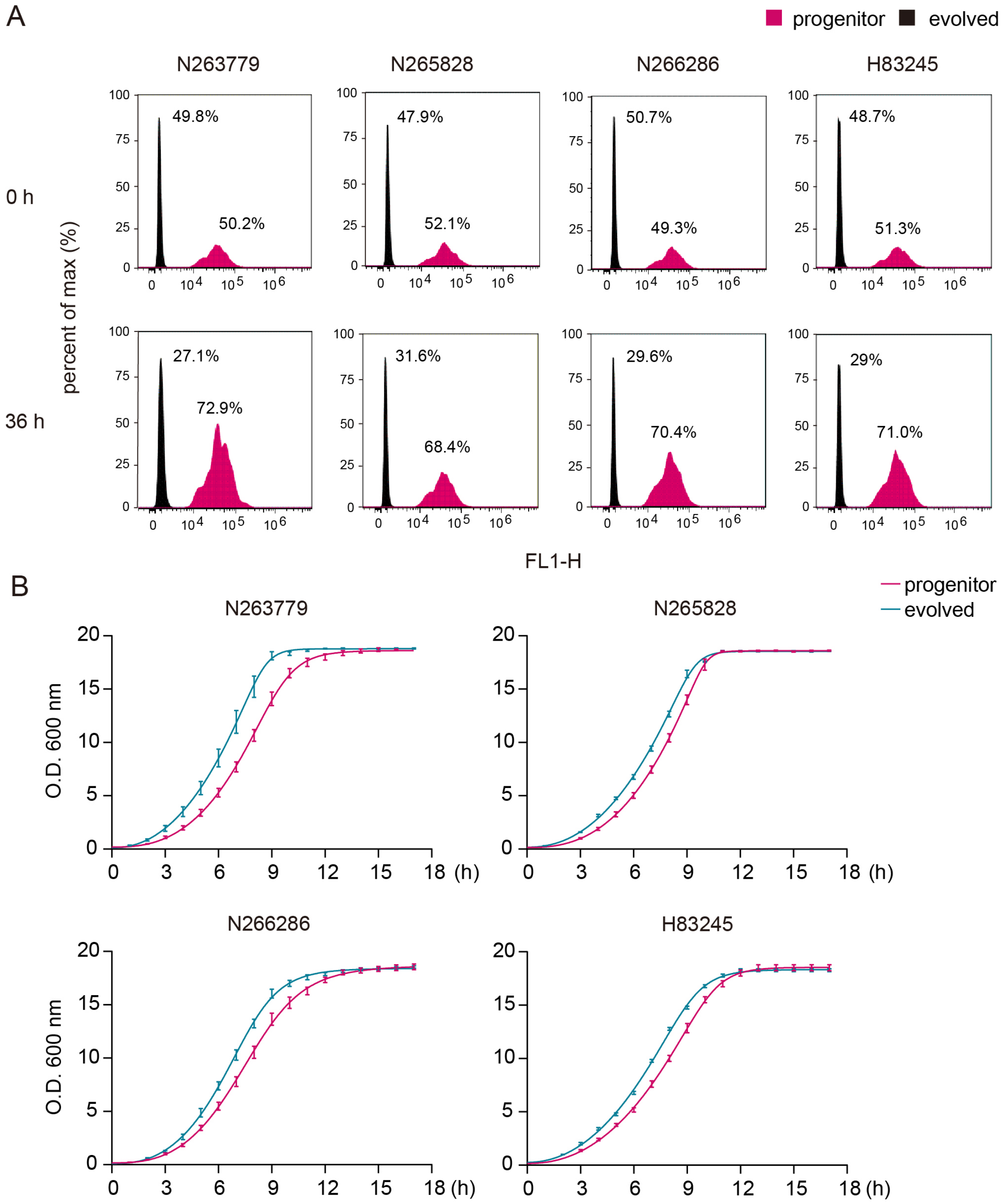

Fig. S6 ***In vitro*** **evolution disfavors growth upon starvation but confers a fitness benefit in nutrient-rich medium at 37°C.** (A) Progenitor cells carrying Hsp90-GFP and *in vitro* evolved cells were mixed as 1:1 ratio and were then incubated in nutrient-poor medium (0.85 g/L YNB, 0.02 g/L L-Tryptophan, 0.1% glucose) for 36 h at 37°C. The resulting cells were subjected to flow cytometry analysis detecting Hsp90-

GFP fluorescence. Representative results of three independent experiments are shown.

(B) The change in optical density over time as a measure of growth was monitored in liquid YPD of progenitor and evolved cells at 37°C. n = 3 biologically independent samples.

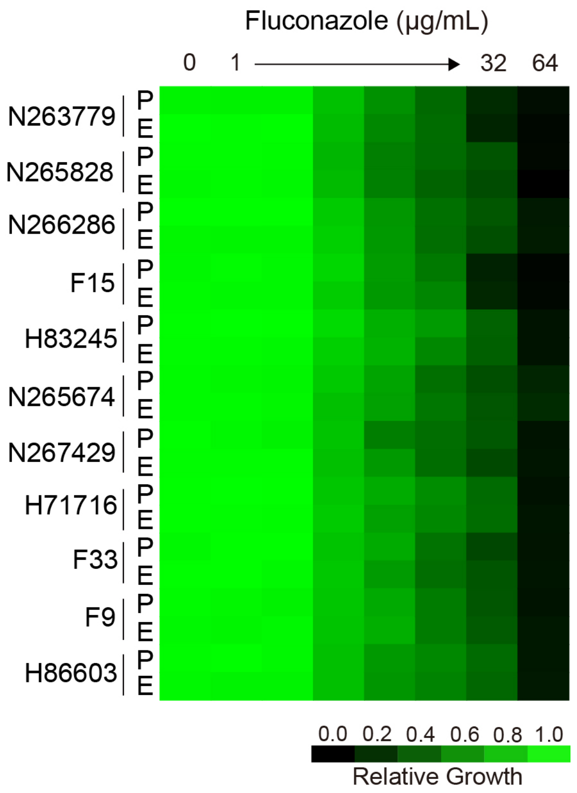

Fig. S7 *In vitro* evolution has no effect on fluconazole sensitivity.
